# Primary purulent bacterial pericarditis due to *Streptococcus intermedius* in an immunocompetent adult: a case report

**DOI:** 10.1186/s13256-018-1570-x

**Published:** 2018-02-05

**Authors:** Mohammad Saud Khan, Zubair Khan, Bhavana Siddegowda Banglore, Ghattas Alkhoury, Laura Murphy, Claudiu Georgescu

**Affiliations:** 1 Department of Internal Medicine, University of Toledo Medical Center, 3000 Arlington Avenue, Mail Stop 1150, Toledo, Ohio 43614 USA; 2 Department of Cardiovascular Medicine, University of Toledo Medical Center, Toledo, Ohio USA; 3 Department of Infectious Diseases, University of Toledo Medical Center, Toledo, Ohio USA

**Keywords:** Primary bacterial pericarditis, Acute purulent pericarditis, *Streptococcus intermedius*, Cardiac tamponade

## Abstract

**Background:**

Acute purulent bacterial pericarditis is of rare occurrence in this modern antibiotic era. Primary involvement of the pericardium without evidence of underlying infection elsewhere is even rarer. It is a rapidly progressive infection with high mortality. We present an extremely rare case of acute purulent bacterial pericarditis in an immunocompetent adult patient with no underlying chronic medical conditions.

**Case presentation:**

A 33-year-old previously healthy white man presented with the complaints of chest pain and dyspnea. He was diagnosed as having acute pericarditis and was discharged home on indomethacin. Over a period of 2 weeks, his symptoms worsened gradually and he was readmitted to our hospital. He was found to have large pericardial effusion with cardiac tamponade. An urgent pericardiocentesis was done with drainage of 550 ml of purulent material. Cultures grew *Streptococcus intermedius* confirming the diagnosis of acute purulent bacterial pericarditis. No other focus of infection was identified on imaging workup suggesting primary infection of the pericardium. His clinical course was complicated by development of constrictive pericarditis for which he underwent surgical pericardiectomy. He received a total of 7 weeks of intravenously administered antibiotics with complete clinical recovery.

**Conclusions:**

Acute purulent bacterial pericarditis, although rare, should always be kept in mind as a possible cause of pericarditis. Early recognition and prompt intervention are important for a successful outcome.

## Background

Bacterial pericarditis is a rapidly progressive infection with high mortality. It is rare in the modern antibiotic era and the majority of cases occur in immunocompromised individuals or in individuals with underlying disease of the pericardium [1, 2]. Bacterial pericarditis usually occurs as a secondary infection by contiguous spread from surrounding intrathoracic focus of infection or by hematogenous spread from distant focus of infection [2, 3]. Primary involvement of the pericardium without evidence of underlying infection elsewhere is very rare. We present a case of a 33-year-old immunocompetent previously healthy adult patient who was diagnosed as having primary purulent acute bacterial pericarditis caused by *Streptococcus intermedius*.

## Case presentation

A 33-year-old white man presented to our hospital with sudden onset pleuritic chest pain and dyspnea of 1 day’s duration. The chest pain started when he was lifting a heavy trash bag and described the pain as sharp, constant, and radiating to his back. He also complained of diffuse body aches and chills but denied any fever, cough, hemoptysis, or weight loss. He denied any history of dental caries, recent travel, or exposure to sick contacts. He had no significant past medical history and was not taking any routine medications. He smoked half a pack of cigarettes a day for the past 10 years and denied any alcohol or illicit drug use. He worked as a waste collector in a garbage disposal firm.

**Fig. 1:**
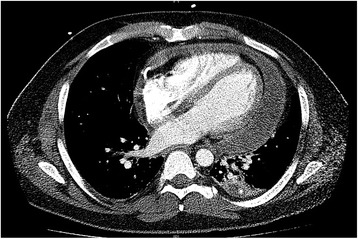
Axial computed tomography scan of chest showing pericardial effusion

At the time of presentation, he was: alert; oriented in time, place, and person; afebrile with a temperature of 37.06 °C (98.7 °F); tachycardic (heart rate of 110 beats/minute); and tachypneic (respiratory rate of 18/minute) with a blood pressure of 126/78 mmHg. An oral examination revealed normal dentition. A cardiopulmonary examination showed normal S1 and S2 with no murmurs and clear lung fields to auscultation bilaterally. An abdominal examination revealed a soft, non-tender abdomen with no organomegaly. A neurological examination showed intact cranial nerves and sensory system, and his muscle strength was 5/5 in all limbs with normal tone. Deep tendon reflexes were normal. An initial laboratory workup showed mild leukocytosis with white blood cell (WBC) count of 13,700 cells/mm^3^, elevated inflammatory markers of erythrocyte sedimentation rate (ESR) 48 mm/hour and C-reactive protein (CRP) 84 mg/dl, and moderately elevated serum transaminases of aspartate aminotransferase (AST) 634 U/L and alanine aminotransferase (ALT) 326 U/L. A basic metabolic panel was within normal limits. Three sets of cardiac enzymes done 8 hours apart were normal. An electrocardiogram was obtained which showed sinus tachycardia and diffuse ST segment elevations. A chest radiograph showed normal bilateral lung fields and normal cardiac silhouette. Computed tomography (CT) of his chest (Fig. 1) ruled out aortic dissection but showed pericardial effusion. A transthoracic echocardiogram showed normal left ventricular systolic function with ejection fraction of 55% and a small pericardial effusion with no signs of tamponade physiology.

**Fig. 2:**
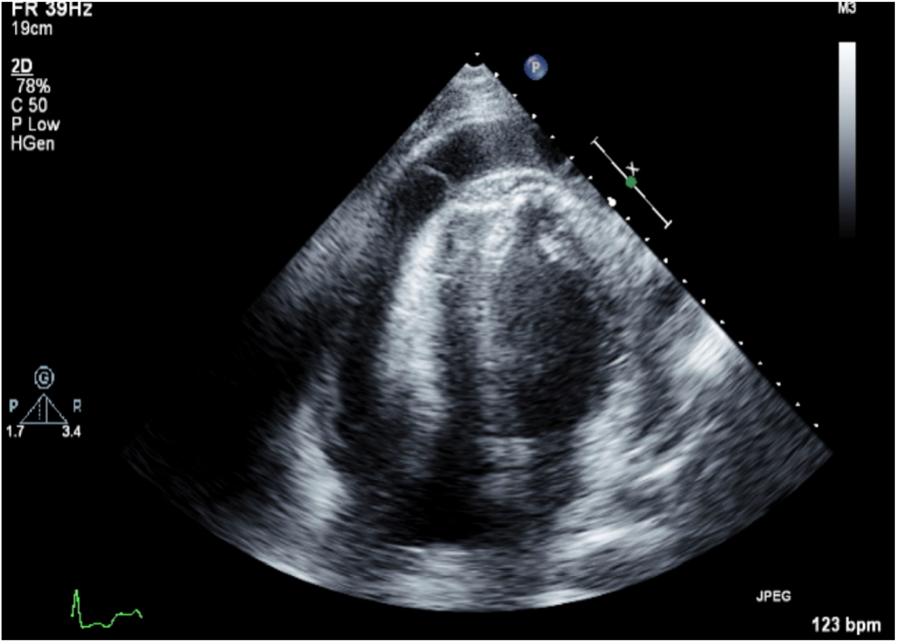
Transthoracic echocardiogram (four chamber view) showing pericardial effusion with presence of internal septations

He was diagnosed as a case of acute pericarditis with minimal pericardial effusion. He was discharged home on indomethacin and was asked to follow up in the out-patient clinic. Two weeks later, when he was seen in our out-patient clinic, he complained of worsening dyspnea and chest pain. He was readmitted to hospital and an urgent transthoracic echocardiogram was obtained, which showed moderate to large pericardial effusion and tamponade physiology with right ventricular diastolic collapse and dilated inferior vena cava (Fig. 2). An emergency pericardiocentesis was done and approximately 550 ml of purulent pericardial fluid was drained. The pericardial fluid was sent for culture, cell analysis, and cytology. Pericardial fluid WBC count was 15,376 cells/mm^3^ with 98% segmented neutrophils. A Gram stain of the pericardial fluid showed Gram-positive cocci in chains. He was started on broad-spectrum antibiotics with intravenously administered vancomycin (dosed on trough concentration) and piperacillin-tazobactam (3.375 g every 8 hours). Pericardial fluid cultures grew alpha hemolytic streptococci which were characterized as *S. intermedius* by matrix-assisted laser desorption/ionization time-of-flight mass spectroscopy (MALDI-TOF MS). *S. intermedius* was susceptible to penicillin and ceftriaxone. Anaerobic, fungal, and acid-fast bacilli cultures were negative. Pericardial fluid cytology was also negative for any malignant cells. Extensive workup with CT abdomen, CT chest, urine analysis, urine culture, and blood culture to look for source of infection failed to identify any other focus of infection. Human immunodeficiency virus (HIV) and tuberculosis QuantiFERON testing were negative. Antibiotics were deescalated to intravenously administered ceftriaxone (2 g every 24 hours) as per culture sensitivity.

**Fig. 3:**
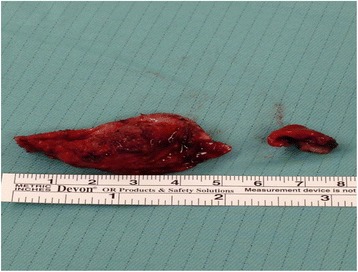
Gross pathological specimen of pericardium after pericardiectomy

His hospital course was complicated with the development of a pulmonary embolism for which he was started on an intravenously administered heparin infusion. However, within a few days of starting heparin, there was a steady decline in his platelets count. Heparin-induced thrombocytopenia (HIT) was suspected and heparin infusion was stopped. Our patient was started on argatroban infusion at a rate of 140 mcg/minute. HIT antibodies and serotonin release assay were sent which came back positive. He was subsequently transitioned to orally administered warfarin with target international normalized ratio (INR) range of 2 to 3. However, with the course of time, he did not have complete resolution of symptoms with persistence of chest pain and dyspnea. Hemodynamic and echocardiographic features were consistent with development of constrictive pericarditis. Surgical pericardiectomy with median sternotomy approach was performed which revealed thickened pericardium (Fig. 3). He reported improvement in his symptoms after pericardiectomy. He was discharged home in a stable condition and received a total of 7 weeks of intravenously administered ceftriaxone. Six months post-discharge he reported complete resolution of his symptoms. A repeat echocardiography was normal and showed resolution of pericardial effusion.

## Discussion

We report a case of primary purulent acute bacterial pericarditis in an immunocompetent previously healthy adult patient caused by *S. intermedius*. Our patient presented with symptoms of dyspnea and chest pain, which gradually progressed over the course of 2 weeks. He was found to have large purulent pericardial effusion with cardiac tamponade and was managed with emergent pericardiocentesis and intravenously administered antibiotics. Further, the disease course was complicated with development of constrictive pericarditis, which was successfully managed with pericardiectomy. Acute pericarditis can be caused by a wide variety of etiologies, which can be infectious or noninfectious [4]. Possible causes include connective tissue disorders, malignancies, radiation, cardiac injury, uremia, and infections (including viral, bacterial, and fungal etiologies) [4, 5]. In a majority of cases (80 to 90%), the cause of pericarditis is not identified [5, 6]. These cases are considered to be idiopathic, most likely due to an undetected underlying virus [5, 6]. Bacterial pericarditis is a rare cause of acute pericarditis in the modern antibiotic era. The reported incidence is < 1% of all cases of pericarditis [7, 8]. The most common organisms implicated are *Streptococci*, *Staphylococci*, *Haemophilus*, and *Mycobacterium tuberculosis* [3, 9].

Bacterial pericarditis usually occurs as a secondary infection by contiguous spread from surrounding intrathoracic infection, including extension from pulmonary, myocardial, and subdiaphragmatic site of infection or by hematogenous dissemination from a distant infection elsewhere in the body [2, 3]. Among these, direct extension from lung or pleura (pneumonia and pleural empyema) accounts for the majority of cases [3, 10]. Very rarely, it can occur as a primary infection without evidence of underlying infection elsewhere. The common predisposing conditions for bacterial pericarditis are immunosuppression, malignancies, preexisting pericardial effusion, alcoholism, uremia, chest trauma, cardiac and thoracic surgery, and insertion or use of catheters for draining pericardial fluid [9, 10]. Purulent pericarditis is a serious manifestation of bacterial pericarditis characterized by the presence of frank pus in the pericardial cavity. It is an acute fulminant disease with rapid progression. It is associated with high mortality and a large number of cases are identified after death [2]. If not identified and treated promptly the mortality rates can be as high as 100% [11]. Even with treatment, the rate of complications and death is high, with the mortality rate approaching 40% [3, 9]. Death is most likely secondary to cardiac tamponade, constriction, and sepsis [3, 9].

Clinical recognition of bacterial pericarditis can be difficult as classical manifestations of acute pericarditis such as chest pain, pericardial friction rub, and pulsus paradoxus may be absent and a patient may present with nonspecific signs and symptoms of infection. Fever is present in almost all of the patients [3]. Chest pain, which can be pleuritic or nonpleuritic, is seen in 25 to 37% of patients [3, 10]. Pericardial friction rub and pulsus paradoxus are seen in less than 50% of patients [3, 10]. Laboratory workup may show evidence of systemic inflammation with leukocytosis, and elevated CRP and ESR [9]. Elevated troponins are seen in approximately 50% of cases [12]. Chest radiograph usually shows cardiomegaly with abnormal cardiac silhouette. Pulmonary infiltrates, pleural effusion, and mediastinal widening may be identified as well [9]. Electrocardiogram findings of acute pericarditis are present in a majority of patients; however, in 10 to 35% of cases findings may be normal [9, 10]. Echocardiogram is the most sensitive test and shows presence of fluid in the pericardial cavity in almost all the patients. However, it is not possible to differentiate purulent fluid collections from other causes of acute pericarditis based on echocardiogram alone. If purulent pericarditis is suspected an urgent pericardiocentesis should be performed for diagnostic and therapeutic indications and fluid should be sent for cell count, Gram stain, culture, and fungal and acid-fast stain. If tuberculous pericarditis is suspected, then performing polymerase chain reaction and adenosine deaminase activity assays on fluid increase the diagnostic yield [13]. Medical therapy includes immediate initiation of broad-spectrum antibiotics with antistaphylococcal agent and an aminoglycoside, which can be followed by 4 weeks of bactericidal antibiotic as per culture and sensitivities. For critically ill patients the empiric antibiotic should include vancomycin, third generation cephalosporin, and a fluoroquinolone [9]. Surgical pericardiectomy is indicated in selected patients with incomplete resolution and complications of the disease.

Our case is an extremely rare case of primary purulent acute bacterial pericarditis in a previously healthy adult individual with no immunocompromising or chronic medical condition, caused by *S. intermedius. S. intermedius* is a member of the *Streptococcus anginosus* group. The *S. anginosus* group (also called *Streptococcus milleri* group) is a subgroup of viridans streptococci that includes three streptococcal species: *S. anginosus*, *S. intermedius*, and *Streptococcus constellatus*. Although these organisms are a part of normal commensal of oral cavity, gastrointestinal tract, and genitourinary tract, they are capable of causing various pyogenic infections and abscess formation [14]. They have been associated with abscess formation in the liver, abdomen, brain, and lung but rarely cause purulent pericarditis.

From a search of the medical literature, we identified a total of 22 cases of bacterial pericarditis caused by *S. anginosus* (*milleri*) group from 1984 to 2017 [3, 15–34]. Out of these, five cases were attributed to *S. intermedius* [18, 28, 31, 34]. To the best of our knowledge, this is the sixth reported case of bacterial pericarditis caused by *S. intermedius*. In our case, the source of *S. intermedius* infection was unclear and development of pericarditis was spontaneous without evidence of any other infective focus elsewhere. *S. intermedius* is a part of normal microbial flora of human oral cavity as discussed earlier. We believe that the most likely etiology of infection in our case was transient bacteremia from a mucosal breach in our patient’s oral cavity with hematogenous spread and seeding of bacteria in pericardial cavity leading to purulent pericarditis.

## Conclusions

Acute purulent bacterial pericarditis, although rare, should always be kept in mind as a possible cause of pericarditis. As this disease has a rapidly progressive fulminant course, early recognition and prompt intervention are critical for a successful outcome.
